# *In vitro* evaluation of *Spirulina platensis* extract incorporated skin cream with its wound healing and antioxidant activities

**DOI:** 10.1080/13880209.2017.1331249

**Published:** 2017-05-29

**Authors:** Seda Gunes, Sedef Tamburaci, Meltem Conk Dalay, Ismet Deliloglu Gurhan

**Affiliations:** a Graduate Program of Biogineering, İzmir Institute of Technology, Izmir, Turkey;; b Department of Bioengineering, Ege University, Faculty of Engineering, Izmir, Turkey

**Keywords:** Cyanobacteria, crude extract, phycocyanin, cytotoxicity, genotoxicity, wound scratch assay

## Abstract

**Context:** Algae have gained importance in cosmeceutical product development due to their beneficial effects on skin health and therapeutical value with bioactive compounds. *Spirulina platensis* Parachas (Phormidiaceae) is renowned as a potential source of high-value chemicals and recently used in skincare products.

**Objective:** This study develops and evaluates skin creams incorporated with bioactive *S. platensis* extract.

**Materials and methods:***Spirulina platensis* was cultivated, the aqueous crude extract was prepared and *in vitro* cytotoxicity of *S. platensis* extract in the range of 0.001–1% concentrations for 1, 3 and 7 d on HS2 keratinocyte cells was determined. Crude extracts were incorporated in skin cream formulation at 0.01% (w/w) concentration and *in vitro* wound healing and genotoxicity studies were performed. Immunohistochemical staining was performed to determine the collagen activity.

**Results:** 0.1% *S. platensis* extract exhibited higher proliferation activity compared with the control group with 198% of cell viability after 3 d. Skin cream including 1.125% *S. platensis* crude extract showed enhanced wound healing effect on HS2 keratinocyte cell line and the highest HS2 cell viability % was obtained with this concentration. The micronucleus (MN) assay results indicated that *S. platensis* extract incorporated creams had no genotoxic effect on human peripheral blood cells. Immunohistochemical analysis showed that collagen 1 immunoreactivity was improved by increased extract concentration and it was strongly positive in cells treated with 1.125% extract incorporated skin cream.

**Conclusions:** The cell viability, wound healing activity and genotoxicity results showed that *S. platensis* incorporated skin cream could be of potential value in cosmeceutical and biomedical applications.

## Introduction

The skin is the body’s first line of defence against infectious organisms and physical damage. It plays a critical role in controlling body temperature and its aging process affects the whole body. Thus the skin is an ideal model for studying mechanism of aging because of its simplicity to observe. Many of the skin changes associated with skin aging (changes in pigmentation, shallowness and deep wrinkling) generally result from sun exposure and categorized as intrinsic (natural skin aging) and extrinsic aging (photo aging) (Simo et al. 2014). Studies concerning skin aging stated that acute exposure of human skin to ultraviolet radiation (UVR) causes oxidation of cellular biomolecules. UVR leads to direct or indirect DNA damage and activates cell surface receptors of keratinocytes and fibroblasts in the skin, which causes breakdown of collagen in the extracellular matrix (ECM) and inhibition of new collagen synthesis. This oxidation results in a depletion of endogenous antioxidants. Thus, it can be prevented by prior antioxidant treatment. The antioxidative defence of the skin depends on the synergistic effect of different antioxidants including vitamins (vitamin E isoforms, vitamin C), nutritive factors and endogenous enzymes (GSH peroxidase (GPx), superoxide dismutase (SOD) and catalase). There are two mechanisms in free radical natural skin defence: enzymatic defence (glutathione peroxidase and superoxide dismutase) and non-enzymatic defence (vitamin C, tocopherols and other food-derived antioxidants) (Helfrich et al. 2008; Pandel et al. 2013).

Focusing on bioproducts, recent trends in biomedical and cosmeceutical applications from natural sources suggest that algae are a promising group producing novel biochemically active substances such as antioxidants, pigments, unsaturated lipids, UV-screens and vitamins (Cardozo et al. 2007). These bioactive compounds can be applied to creams, lotions and ointments (Helfrich et al. 2008; Kim et al. 2008). The major product groups of cosmeceuticals consist of anti-aging products because antioxidants play a large role in body maintenance and repairing system.

Recently, there has been a great deal of interest in cosmetic and biomedical applications (e.g., anti-aging cream, refreshing or regenerant care products, emollient and as an anti-irritant in peelers, wound healing patches) regarding active ingredients including dietary fibre, ω-3 fatty acids, essential amino acids, vitamins (A, B, C, E), minerals, botanical extracts and antioxidants from marine microalgae. They are also known as producers of secondary metabolites which are biologically active compounds with antiviral, antibacterial, antifungal, anti-inflammatory and anticancer activities. These bioactives obtained from microalgae and cyanobacteria have various beneficial effects on human skin health by skin, hair and nail health promotion at cellular levels. Extracts of microalgae promote healing in skin damages and inhibit the inflammation process. Some species of microalgae such as *Spirulina* (syn. *Arthrospira*)*, Pseudanabaena, Chlorella, Haematococcus, Dunaliella* and *Porphyridium* have a great importance as colorants and additives in the cosmetic industry. *Spirulina* and *Chlorella* are established in the skin care market as commercially available products; repairing the signs of early skin aging, exerting a tightening effect and stimulating collagen synthesis, preventing stria formation and wrinkle reduction (Spolaore et al. 2006; Kim et al. 2008). Also, marine algae have been indicated to be a good source of photoprotective agents with their mycosporine-like amino acids (MAAs), carotenoids and polyphenols. They are represented in sun protection creams and hair care products with these UV protective compounds (Pulz & Gross 2004; Kim 2011).

*Spirulina platensis* Parachas (Phormidiaceae) (syn. *Arthrospira platensis*) is rich in phycocyanin and this pigment has been widely used as a natural blue colorant for the cosmetic additive. The biochemical composition of *Spirulina* indicates that it has high nutritional and nutraceutical value due to its content of a wide range of essential nutrients, such as provitamins, minerals, proteins, polyunsaturated fatty acids such as γ-linolenic acid and phenolic acids, tocopherols and β-carotene which are known to exhibit antioxidant properties (Miranda et al. 1998; Hirata et al. 2000; Colla et al. 2007). *Spirulina platensis* is considered as nature’s richest source of vitamin B12 and have high amino acid content (62%), possessing antiviral, anticancer, hypocholesterolemic, anti-diabetic, antioxidant, anti-inflammatory and anti-metastasis activities. These properties make *S. platensis* extract a potential pharmaceutical for biomedical applications. *In vitro* studies show that *S. platensis* enhances cell nucleus enzyme activity and DNA repair synthesis with its polysaccharide content and in a recent study, the aqueous extract of *S. platensis* showed a protective effect against apoptotic cell death in the cause of free radicals (Estrada et al. 2001; Joventino et al. 2012). Therefore, *Spirulina* extracts could be incorporated into biomaterials due to its ECM-like bioactive molecules to form tissue-like matrices and thus can mimic ECM (Kim et al. 2012).

According to FDA, cosmeceuticals are applied to the human body without affecting the structure and functions of the body. Some chemicals in the cosmetic products may penetrate the skin and can cause allergy, genotoxicity and cytotoxicity (Maithili et al. 2015). Genotoxicity testing is important in the evaluation of cosmeceuticals and chemicals wherein cosmetic formulations, for EU regulations. There is a growing interest in the alternative *in vitro* tools to replace animal tests, especially in the light of regulations such as the 7th amendment to the Cosmetics Directive (EU 2003) and national animal protection laws. Therefore, in recent years, the use of *in vitro* models is growing interest and could be an alternative to animal testing for the evaluation of genotoxicity of cosmetic products (Tweats et al. 2006; Pfuhler et al. 2014; Speit 2009).

This study develops natural skin creams incorporated with bioactive *S. platensis* extract and evaluate the *in vitro* cytotoxic, genotoxic effects for the safety of consumer products and wound healing activity. Extract incorporated skin creams were evaluated in both cytotoxicity and genotoxicity tests, while *Spirulina*’s positive effect on skin cell proliferation was determined by *in vitro* scratch assay as a wound healing model.

## Materials and methods

### Organism and culture conditions

*Spirulina platensis* was provided by Ege University, Bioengineering Department, Microalgae Culture Collection (EGE-MACC 38). Human keratinocyte cell line (HS2) and human fibroblast cell line (L929) which were used in cytotoxicity and wound healing assays were provided by Animal Cell Culture Collection (HUKUK, Sap Institute, Ankara, Turkey). Human peripheral blood culture used in genotoxicity studies was obtained from healthy individuals (Ege University, Faculty of Medicine Research Ethics Committee with approval number: 09-3/10).

*Spirulina platensis* was cultivated in 2 L culture bottles containing modified Zarrouk’s medium at pH 9.80–10.0 for 10 d (Zarrouk 1966; Morist et al. 2001). Zarrouk’s medium was modified and the chemical composition is depicted in Table 1. Inoculum concentration was 0.15 g dry weight/L as referred by Pelizer et al. (2003). The cultivations were carried out at 25 °C, 2000 lux of luminance provided by fluorescent lamps.

**Table 1.** Composition of modified Zarrouk medium.

Ingredients		Amount (g/L)	
NaCl		1	
MgSO_4_·7H_2_O		0.2	
CaCl_2_		0.04	
FeSO_4_·7H_2_O		0.01	
EDTA		0.08	
NaNO_3_		2.5	
K_2_HPO_4_		0.5	
K_2_SO_4_		1	
NaHCO_3_		16.8	
Na_2_CO_3_		7.6	
A_5_		1 mL	
B_6_		1 mL	
A_5_ composition		B_6_ composition	
Ingredients	Amount (g/L)	Ingredients	Amount (g/L)
H_3_BO_3_	2.86	NH_4_VO_3_	0.0022
MnCl_2_·4H_2_O	1.81	KCr(SO_4_)·12H_2_O	0.096
ZnSO_4_·7H_2_O	0.222	NiSO_4_·6H_2_O	0.045
CuSO_4_·5H_2_O	0.075	Na_2_WO_4_	0.018
MoO_3_·H_2_O	0.015	TiO_2_	0.016

In proliferation and wound healing assays, human keratinocyte cells (HS2) and human fibroblast cells (L929) were cultured in DMEM/F12 culture medium (ALDRICH, Sigma, St. Louis, MO) contained 10% foetal bovine serum, 1% l-glutamine (10 μg/mL) and 10 μg/mL penicillin/streptomycin in 37 °C, CO_2_ incubator. Additionally, 1% sodium pyruvate was added in culture medium for L929 cells.

Peripheral blood lymphocyte culture was used in micronucleus (MN) assay. Peripheral blood cells were cultured in RPMI 1640 culture medium contained 20% foetal bovine serum, 20 μg/mL penicillin/streptomycin and 1% l-glutamine, in 37 °C, CO_2_ incubator. Phytohemoagglutinin (PHA, Biochrom, Berlin, Germany) was used for stimulating cells to enter mitosis. Mitomycin C (Panreac AppliChem, Darmstadt, Germany) was used as a positive control and cytochalasin B (Sigma, St. Louis, MO) was added for blocking cytokinesis in MN assay.

### *Spirulina platensis* growth

*Spirulina platensis* cultures were evaluated morphologically by microscopic observation (Olympus CH40, Center Valley, PA) during cultivation. Samples were taken at indicated times, and following growth parameters were measured immediately. The increase in growth and biomass was evaluated using dry weight determination and turbidity. Dry weight was determined by filtering culture samples with Whatman GF/C filters and drying the cell mass at 105 °C for 2 h. Turbidity was measured by recording the absorbance at 750 nm in order to determine cell concentration. Measurements were carried out in triplicate.

### Crude extract preparation and biochemical characterization

Cultured cells were harvested by centrifugation for 30 min at 5000 rpm and washed three times with a 1 × PBS solution. Centrifugated biomass (1 g) was added into sonication buffer for cell disruption (10 mM potassium phosphate with 0.1 mM EDTA at pH 7.8). The samples were sonicated with an ultrasonic homogenizer (Bandelin Electronic HD-2070, Berlin, Germany) for 10 min. *Spirulina* crude extracts were obtained by the method of Minkova et al. (2003).

Protein amount of *Spirulina* crude extract was determined spectrophotometrically by modified Lowry method (Bensadoun & Weinstein 1976). Colorimetric Lowry protein assay is based on the biuret reaction by subsequent reaction with the Folin phenol reagent (Folin–Ciocalteu reagent). Bovine serum albumin (BSA) was used as a standard in the assay (Lowry et al. 1951).

Phycocyanin content of the crude extract was determined by the method of Boussiba and Richmond (1979) by spectrophotometrically at 620 nm.

Superoxide dismutase (SOD) activity of *S. platensis* was determined by xanthine/xanthine oxidase (X–XOD) method using RANSOD® enzyme kit (RANDOX Laboratories Ltd, Charles Town, WV). This method employs reaction of xanthine with xanthine oxidase (XOD) to generate superoxide radicals which react with 2-(4-iodophenyl)-3-(4-nitrophenol)-5-phenyltetrazolium chloride (I.N.T.) to form a red formazan dye. The inhibition of the rate of reduction of INT under the conditions of the assay refers to inhibition % values. The assay was carried out at 37 °C using double-beam spectrophotometer (Shimadzu Digital, UV-160A, Shimadzu Corp, Tokyo, Japan) at 505 nm with heat jacket.

### *In vitro* cytotoxicity of crude extracts and cream ingredients

Cytotoxicity assay of *S. platensis* extracts was performed to determine the extract concentration to be incorporated into skin cream for further *in vitro* wound scratch and genotoxicity assays. Cytotoxicity of *S. platensis* extracts was evaluated by the MTT [3-(4,5-dimethylthiazol-2-yl)-2,5-diphenyl tetrazolium bromide] assay. HS2 cells were seeded at 10^5^ cells/well in 24-well plates and incubated at 37 °C in a 5% CO_2_ incubator. Following incubation, cells were treated with different concentrations of *S. platensis* extract (1, 0.75, 0.50, 0.1, 0.05, 0.01, and 0.001%) for 1, 3 and 7 d with each extract. HS2 cells were cultivated with DMEM/F12 medium supplemented with 10% foetal calf serum in an atmosphere of 5% CO_2_ at 37 °C. After the incubation time, the extent of reduction of MTT to formazan with cells was quantified by using the microplate spectrophotometer system at 570 nm (Molecular Devices, Versamax 190, Silicon Valley, CA). The experiment was carried out in triplicate. In order to evaluate the biocompatibility of each ingredient incorporated into the cream formulation, *in vitro* cytotoxicity assay (MTT) was performed. Cytotoxicity of cream ingredients on HS2 cell line was carried out in the same procedure asserted above.

### Incorporation of *S. platensis* extract into skin creams

*Spirulina platensis* extract incorporated skin creams were developed by Dalan Chemistry Company, Izmir, Turkey. *In vitro* cytotoxicity testing is generally recommended for *in vitro–in vivo* correlation. Keratinocyte culture models have been suggested for a better correlation of *in vitro* toxicity testing with *in vivo* irritation potential. However, *in vitro* tests may not fully reflect the *in vivo* situation; attributed to possible physiologic responses, differences in sensitivities and all possible reaction (Keong & Halim 2009). Gur et al. (2013) showed the proliferative effect of different crude extract ratios (0.50 and 1.25%) for *in vivo* wound healing model. In this study, cytotoxicity of *S. platensis* crude extracts was determined by MTT assay. Crude extracts were incorporated into skin creams with 0.50% and 1.125% ratios based on *in vitro* cytotoxicity assay of crude extracts.

### *In vitro* wound healing, proliferation and migration assay

**Figure 1.**
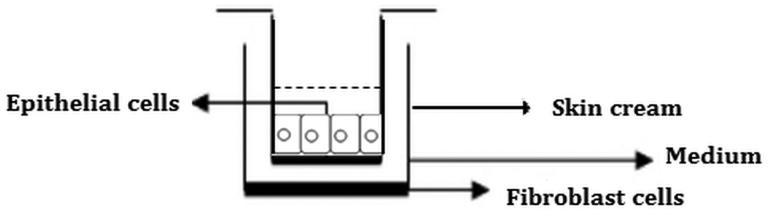
Cell culture insert model.

**Figure 2.**
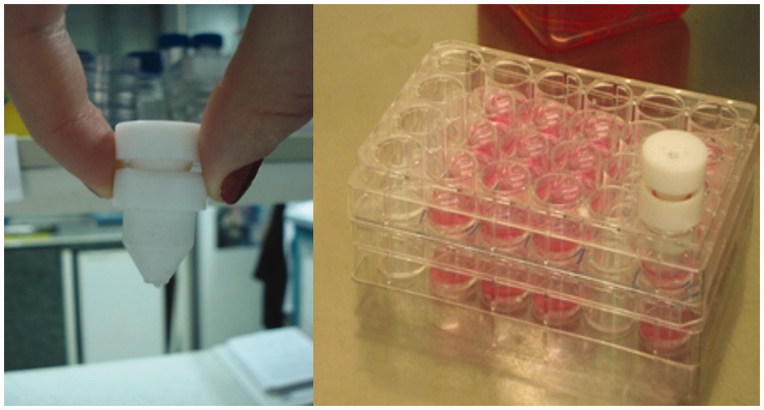
Wound creating apparatus and making a scratch in 24-well plate.

*In vitro* wound healing effect of skin creams including 0.5% and 1.125% *S. platensis* extracts were evaluated by the *in vitro* scratch assay. Wound healing was performed in 24-well plate. In order to mimic skin layer consisting of human fibroblast and keratinocyte cells, cell culture inserts (Nunc, Transwell 24-well insert, Rochester, NY) were used. HS2 cells were seeded in the upper compartment of cell culture inserts for skin cream penetration (Figure 1), while L929 fibroblasts were seeded in lower compartment to observe cell migration. L929 cell line was seeded in 24-well plate and then incubated at 37 °C, 5% CO_2_ for 3 h to provide a confluent monolayer. The wound healing activity was performed by using *in vitro* wound healing method. After incubation, scratches were created on cell layer, as diameter circular zone with a sterile Teflon bar apparatus that removes cells (Figure 2). This apparatus was developed by Ege University, Faculty of Dentistry, in order to obtain homogenous scratches (Arıkan et al. 2007). The debris was removed by washing and replaced with a specific medium for the *in vitro* scratch assay (Liang et al. 2007). HS2 cells were seeded in the upper compartment of cell culture inserts and cells were incubated at 37 °C, 5% CO_2_ to obtain confluent monolayer. After incubation, skin creams were transferred onto cell culture inserts which were placed on HS2 cells. Cells were exposed to skin creams for 5 and 10 d. Culture medium was refreshed every other day during the cultivation. Cell control without any skin cream formulation was used as negative control. *In vitro* cell proliferation and wound healing, experiment sets were carried out in triplicate.

### Determination of genotoxicity with MN assay

MN assay was carried out for *in vitro* genetic toxicity determination according to OECD guidelines. Peripheral blood cell culture obtained from a healthy young non-smoking individual was prepared with RPMI 1640 medium (Biochrom, Berlin, Germany). The study was permitted by Ege University, Faculty of Medicine Research Ethics Committee (approval number: 09-3/10) and was performed according to the international rules. Phytohaemagglutinin (PHA) was used as a mitogen for selectively stimulating cells to enter mitosis. Cells were exposed to test agents (cream extracts containing 0.5% and 1.125% *S. platensis* prepared according to ISO 10993-12). Mitomycin C (Panreac AppliChem, Darmstadt, Germany) was used as a positive control. After treatment by cream extracts, cytochalasin B (ALDRICH, Sigma, St. Louis, MO) was added for blocking cytokinesis and cell cultures were grown for a sufficient period to allow chromosomal damage to lead to the formation of micronuclei, bi- or multinucleated interphase cells. Harvested and stained interphase cells were analyzed microscopically for the presence of micronuclei. The slides from the control and the treated cultures were examined under a microscope. A total of 1000 cells were analyzed for each individual and type of culture for MN assay. Cells containing one or more micronuclei were recorded as micronucleated cells. The identification criteria of MN were described by Fenech et al. (2003). All data were expressed as the mean ± standard deviation (SD). However, MN values were not normally distributed. Experiments were carried out in triplicate.

### Immunohistochemical studies

Immunohistochemical staining was performed with avidin–biotin peroxidase complex method (Grawish et al. 2010). Before staining procedure, cell layers were treated with 4% paraformaldehyde for 30 min to perform fixation and washed with phosphate buffer solution (PBS) thrice. Briefly, samples were treated with 3% hydrogen peroxide (Merck, Darmstadt, Germany) for 5 min and washed with PBS three times, then preincubated with 1% Triton-X 100 for permeabilization followed by rinsing with PBS. After 1 h blocking, samples were incubated with anti-collagen 1 (GTX 41285, GeneTex, Irvine, CA) antibody overnight. Samples were treated with secondary antibodies biotinylated IgG and streptavidin (SentiTek HRP Anti-Polyvalent, SHP125, Skytek, Garden City, GA). The peroxidase reaction was performed with 3,30-diaminobenzidine tetrahydrochloride (DAB) chromogen. Finally, specimens were examined by light microscopy (BX40 Olympus, Center Valley, PA).

### Statistical analysis

Data were analyzed statistically using analysis of variance. Statistical significance of the difference between the data pairs was evaluated by one-way ANOVA and it was considered significant at *p* < 0.05.

## Results

### Crude extract preparation and biochemical characterization

*Spirulina platensis* was cultured under the optimum growth conditions for 10 d of cultivation period. SOD activity, protein and phycocyanin contents of crude extracts were determined after cultivation period. *Spirulina platensis* is well known with its high protein content and shows strong antioxidant properties due to its SOD activity. Biochemical analysis indicated that *Spirulina* extract contained 0.27 mg/mL protein and 0.28 mg/mL phycocyanin. In accordance with 40–60% protein content found in this study performed by also Oliveira et al. (1999). *Spirulina platensis* also exhibited strong antioxidant property due to its SOD activity found as 8.0 U/mL in this study.

### *In vitro* cytotoxicity and wound healing activities of crude extracts and cream ingredients

**Figure 3.**
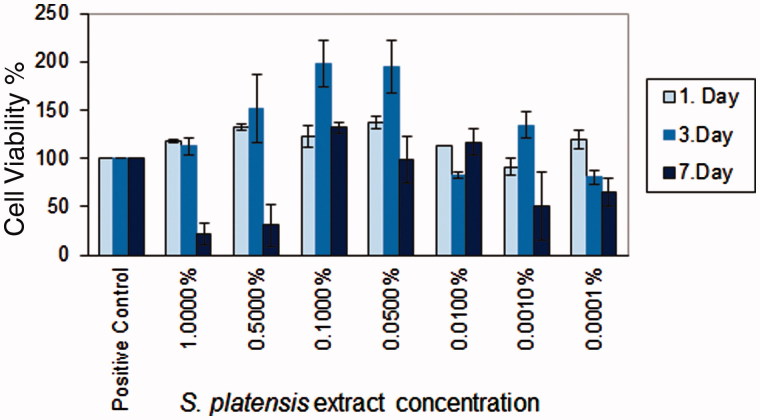
*In vitro* cytotoxicity of *S. platensis* extracts on HS2 keratinocyte cell line.

Cell viability and proliferation effects of the *S. platensis* crude extract at different concentrations were evaluated by MTT assay. It was found that crude extracts did not show the cytotoxic effect and were biocompatible on HS2 cell line (Figure 3). However, crude extracts exhibited a proliferative effect on cultured cells. The crude extract concentrations were ranked according to cell viability as 0.1 > 0.05 > 0.5 > 0.01%. The highest proliferative activity was obtained with *S. platensis* crude extract at the concentration of 0.1% and 0.05%. *Spirulina platensis* extract concentrations were determined according to *in vitro* cytotoxicity results and were incorporated into skin creams. Cell proliferation activity has been attributed to high phycocyanin content. Phycocyanin content of *S. platensis* crude extract was determined as 4.5%. The cell viability results indicated that all tested cream ingredients did not show any cytotoxic effect and the cream formulation was regarded as biocompatible (data not were shown).

**Figure 4.**
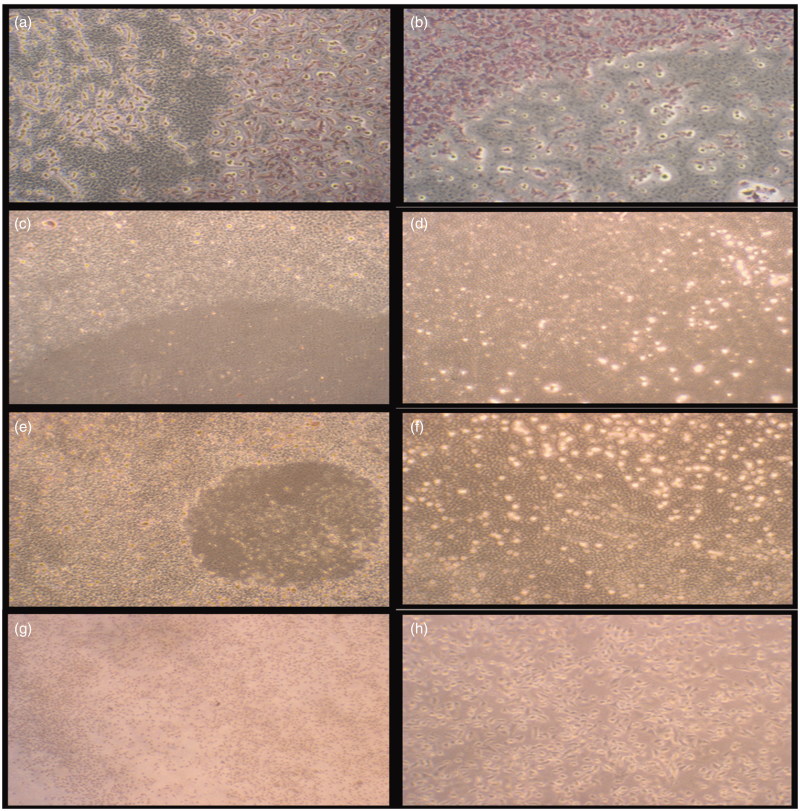
Morphological observation of the wounded edge and centre of HS2 cell cultures. (a,b) Negative cell control (4×, 10×); (c,d) cream control (4×, 10×); (e,f) cream with 0.5% *Spirulina platensis* extract (4×, 10×); (g,h) cream with 1.125% *S. platensis* extract (4×, 10×) cell migration and wound healing for 5 d.

For assessment of *in vitro* wound healing effect of the samples in terms of cell viability and proliferation, both fibroblast and HS2 cells were treated for 5 and 10 d. Cell migration was also evaluated in a negative control and cream control to compare crude extract incorporated creams. Cell proliferation and migration were increased by the addition of crude extract. It could be important to test the effects of both direct contacts with cells and indirect exposure to diffusible components to study cytotoxicity. The wound healing effects of *S. platensis* extract incorporated skin creams on L929 fibroblast cells in 5 and 10 d are represented in Table 2. Data were represented as mean ± standard deviation (SD). In order to observe the proliferation and migration of cells which were exposed to skin cream examined by light microscopy. The negative cell control group was stained with Giemsa, while cells exposed to skin cream could not be stained due to hydrophobic nature of cream. Therefore, microscopy was employed to observe proliferation and migration of cells exposed to skin cream (Figure 4). *In vitro* wound healing activity was observed with ‘scratch assay’ model. The results showed that cell migration in the wound area has been significantly improved with *S. platensis* extract compared with that of the control cream and the cell control group (*p* < 0.05).

**Table 2:** Wound closure effects of negative cell control, skin cream and crude extract incorporated skin cream.

Groups	Wound closure (diameter: 2.5 mm)	Wound closure (%)
Cell control	0.575 ± 0.0138	23
Cream control	0.849 ± 0.111	33.9
0.5% crude extract	1.416 ± 0.074	56.6
1.125% crude extract	1.873 ± 0.217	74.9

The effect of 0.5% and 1.125% *S. platensis* extracts incorporated skin creams on cell migration and proliferation in terms of wound area is depicted in Figure 4. It was found that skin cream with 1.125% *Spirulina* extract was more efficient and exhibited a highest proliferative effect on skin cells.

### Immunohistochemical results

**Figure 5.**
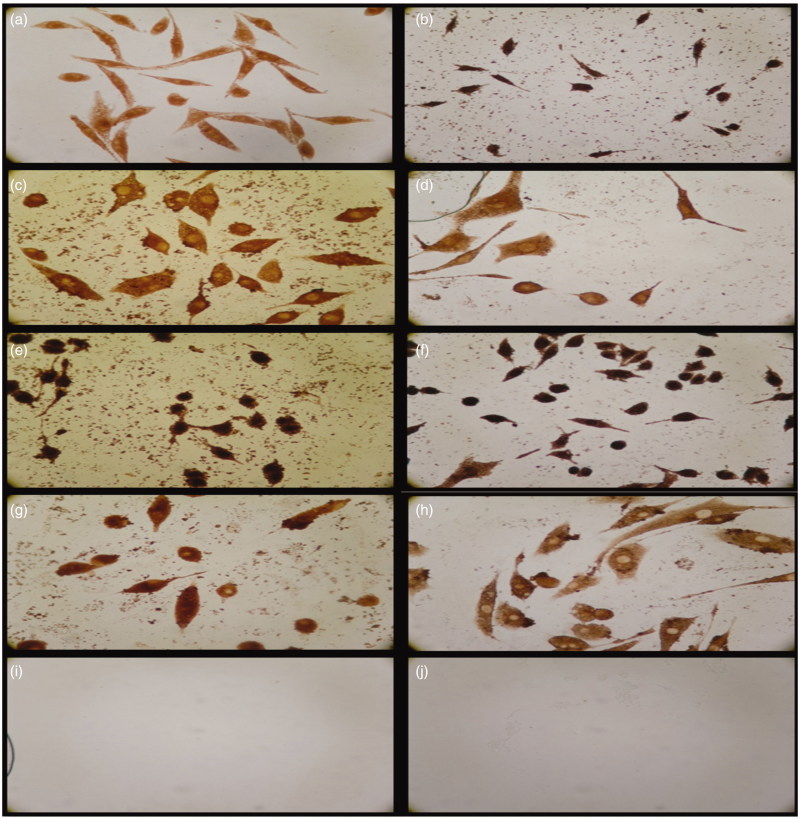
Immunohistochemical staining results of HS2 cell cultures. (a,b) Negative cell control (5 d, 10 d); (c,d) cream with 0.5% *Spirulina platensis* extract (5 d, 10 d); (e,f) cream with 1.125% *S. platensis* extract (5 d, 10 d); (g,h) cream control (5 d, 10 d) and (i,j) IHC control staining (5 d, 10 d).

The immunohistochemical assay was performed on the 14th day of incubation with cream formulations. Fibroblast morphology was observed almost in all test groups. In the immunohistochemical examination, the control cells and skin cream-treated cells showed very weak collagen 1 immunoreactivity. It was found that collagen 1 immunoreactivities of positive control and skin cream-treated cells were weaker than extract incorporated creams treatment. However, collagen 1 immunoreactivity was improved by increased extract concentration and it was strongly positive in cells treated with 1.125% extract incorporated skin cream. Control IHC staining was used as a negative control (Figure 5).

It was revealed that *S. platensis* extracts are also composed of flavonoids, alkaloid and triterpenoids which are known to promote the wound healing process mainly due to their antimicrobial and wound contraction property and increased rate of epithelization (Panigrahi et al. 2011). These results suggest that crude extracts of *Spirulina platensis* have a promising potential to be used in cosmeceutical applications.

### Genotoxicity

**Figure 6.**
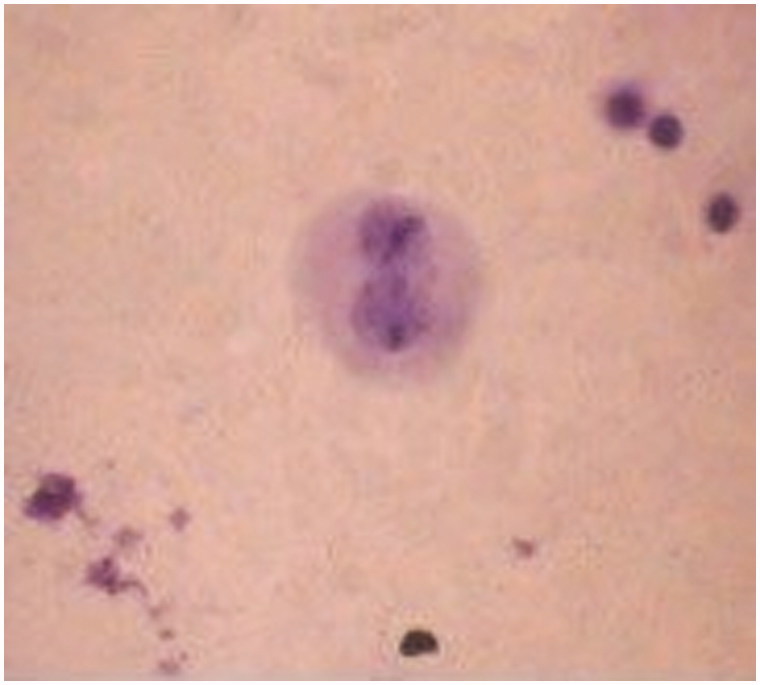
Micronucleus formation during incubation with skin cream extracts.

There is a growing concern that biomedical devices or pharmaceutical products may exert some genotoxic effects, even though they may be cytocompatible. Genotoxicity and mutagenicity testing are important in the assessment of chemicals for EU regulations. Besides, the daily use of cosmeceuticals can cause skin local problems and also systemic effects after absorption via the skin (Maithili et al. 2015). Genotoxic effects will eventually lead to abnormal and reduced cell growth, even if the cells initially appear cytocompatible. MN assay has become an important tool for the evaluation of the genotoxicity of various products. MN assay was performed with control skin cream and creams containing different amounts of *S. platensis* crude extract. The positive control, mitomycin C, induces chromosome damage or damage to the cell division apparatus, whereas negative control does not induce chromosome structural and or numerical aberrations of chromosomes in cultured mammalian somatic cells. Cytochalasin-B (Cyt-B) was added to cultures to block cytokinesis. MN formation was observed by light microscopy. The number of MN was counted and reported as the total number of cells observed. Figure 6 shows the number of micronuclei found at skin cream treated cells under analysis. Binucleated cells per duplicate in cell culture were recorded to evaluate the frequency of cells with one, two or more than two micronuclei. About 92% of negative control cells showed no MN formation. About 98–99.4% of skin cream treated cells were observed without MN formation. Results clearly demonstrated that *S. platensis* extract incorporated creams did not show any mutagenic effect and there was no significant difference between groups according to the MN assay. The effect of skin cream treatment on cells and range of spontaneous percentages of micronucleated cells are reported in Table 3 and Figure 6. In this assay, no significant increase in the number of micronucleated cells was found and the results indicated that MN formation was not observed in skin cream control and skin cream with *S. platensis* crude extract (*p* < 0.05).

**Table 3.** Distribution of micronuclei (MN) in the binucleated (BN) cells scored, total number of MN, mean ± SD.

		Distribution of MN in Nucleated and binucleated (BN) cells (%)			
Group	Cells without micronucleus (%)	1	2	3≥	MN frequency ± SD (%)
Control	92.9	4.6	2.2	0.3	7.1 ± 1.12
Mitomycin C	65.7	20.3	6.7	7.3	34.5 ± 2.8
Skin cream	99.4	0.6	0	0	0.6 ± 0.46
0.5% Extract incorporated cream	98	2	0	0	2 ± 0.57
1.125% Extract incorporated cream	98.7	1.3	0	0	1.3 ± 1.15

In comparison with the negative control, all tested cream sample results showed no statistical difference in MN levels. In conclusion, different extract concentrations in cream formulation did not affect genotoxicity results significantly according to ANOVA. However, cell numbers with and without MN showed a significant difference.

## Discussion

Cyanobacteria are oxygen evolving photosynthetic micro-organisms which produce reactive oxygen species (ROS). ROS damage cellular components such as phospholipids and DNA. Cyanobacteria scavenge ROS with their antioxidative systems. *Spirulina platensis* has two important phycobiliproteins known as phycocyanin and allophycocyanin. Previous studies have shown that phycocyanin has potential antioxidant and anti-inflammatory properties by scavenging peroxyl, hydroxyl and superoxide radicals (Bhat & Madyastha 2001; Estrada et al. 2001; Romay et al. 2003; Bermejo et al. 2008). Besides, in previous studies, the antioxidant potential of the phycocyanin present in *S. platensis* was investigated and results indicated that phycocyanin administration to the rats (5 mg/d) had the significant antioxidant effect to reduce the oxidative damage caused by monosodium glutamate *in vivo* (Bertolin et al. 2011). In our study, the crude extract of *S. platensis* was found to have high protein and phycocyanin content, as expected. Additionally, high SOD activity which is responsible for ROS scavenging was obtained.

In this study*, in vitro* cytotoxicity of *S. platensis* crude extracts, *in vitro* wound healing assay and genotoxicity studies of crude extract incorporated in skin cream formulations were performed. Based on the results, it can be concluded that crude extracts have a proliferative effect on HS2 cell culture. Crude extract concentrations of 0.1% and 0.05% (w/v) showed the highest proliferative activity in comparison with all tested concentrations. In another study, the crude extract of *S. platensis* and C-phycocyanin extracts were compared in terms of wound healing activity and it was found that C-phycocyanin directly enhanced *in vitro* cell proliferation and *in vivo* wound repair (Gur et al. 2013). Since *S. platensis* contains a wide range of other nutrients such as phycocyanin, linolenic acid, β-carotene, vitamins and proteins, the crude extract was used rather than pure compound extracts from *S. platensis*.

*In vitro* wound healing activity of *S. platensis* extract was assessed by *in vitro* wound scratch assay. In wound area, cell migration and proliferation were observed by measuring the radius of the wound area. Results showed that cell proliferation and migration were increased from 23% (control group) to 56.6% and 74.9% by incorporation of 0.5% and 1.125% crude extracts, respectively. Skin cream incorporated with 1.125% *S. platensis* extract exhibited the highest proliferative effect on skin cells. The present investigation reveals that skin creams incorporated with different concentrations of *S. platensis* extracts are capable of producing significant wound healing activity in keratinocyte cells. The natural formulation of skin creams especially with *S. platensis* extract provided stimulatory effect and, therefore, showed a good result in wound healing. Syarina et al. (2015) prepared aqueous, methanolic and ethanolic extracts of *Spirulina* and conducted a wound scratch study on human dermal fibroblast cell line (HDF) with and without *S. platensis* extract treatment. Aqueous extract of *Spirulina* enhanced migration and wound closure of HDF fibroblast cells. Aqueous extract-treated fibroblast cells showed faster cell proliferation and migration in comparison with untreated cells and cells treated with methanol and ethanol extracts (Syarina et al. 2015). *In vitro* scratch assay was performed as a small linear scratch created in the confluent monolayer by gently scraping with pipette tips. As distinct from this scratch assay, different circular apparatus was used in our study to obtain homogenous scratch for quantitative determination of cell migration.

Collagen 1 increase is one of the most important factors in skin regeneration and wound healing process. In order to determine the collagen formation, the immunohistochemical assay was performed. Immunohistochemical assay results showed that collagen 1 immunoreactivities increased with 0.5% and 1.125% extract incorporated skin creams. Increased collagen 1 immunoreactivity was observed in cells treated with 1.125% extract incorporated skin cream. Panigrahi et al. (2011) showed that *S. platensis* extracts exhibited wound healing activity on *in vivo* rat models. In addition, they found flavonoids, alkaloid and triterpenoids as the result of phytochemical analysis of the *Spirulina* extracts. Triterpenoids and flavonoids are known to promote the wound healing process mainly due to their antioxidant and antimicrobial property, which seems to be responsible for wound contraction and increased rate of epithelization.

Due to the fact that chromosomal mutation is crucial in carcinogenesis, determination of DNA damage at the chromosome level is important for genetic toxicology. The MN assay is one of the preferred methods for evaluation of chromosomal damage because this assay enables chromosome loss as well as chromosome breakage (Fenech 2000). Therefore, genotoxic effects of *S. platensis* extracts incorporated in the skin creams were assessed by *in vitro* MN assay. Genotoxicity may cause abnormal and reduced cell growth. Mitomycin C was used as a positive control and cytochalasin-B (Cyt-B) was added to cultures. The number of MN formation was counted and reported as the total number of cells observed. This assay showed that no significant increase in the number of micronucleated cells was found and there was no observed mutagenic effect in the tested creams with and without (skin cream control) *S. platensis* extract (*p* < 0.05). In previous studies, *Arthrospira maxima* known as a different species of *Arthrospira* genus is investigated due to its protective effects against genotoxicity and mutagenicity. Argüelles-Velázquez et al. (2013) examined the effect of *Arthrospira maxima* on the teratogenicity, genotoxicity and DNA oxidation processes induced by cadmium (Cd) by oral administration in mice. Results of this study indicated that *A. maxima* may reduce the genotoxic effects of congenital malformations due to Cd exposure in utero and this effect may arise from its antioxidant activity. Similarly, Chamorro-Cevallos et al. (2014) investigated the possible antimutagenic effects of *Arthrospira maxima* on male and female mice by oral administration. They indicated that *A. maxima* exhibited a protective effect against benzo(α)pyrene induced genetic damage to germ cells in male and female mice.

## Conclusions

The skin is the first line defence for the body against infections and physical conditions such as temperature and radiation. Novel trends tend to multifunctional biomedical and cosmeceutical products with photoprotective, anti-aging and wound healing activities. *Spirulina platensis* extract contains a mixture of proteins and carotenoids which have a synergistic effect on skin cell proliferation, wound healing and tissue regeneration. In this study*, in vitro* cytotoxicity and wound healing effects of *S. platensis* extracts were investigated in order to evaluate the potential usage in biomedical and pharmaceutical area. *In vitro* cell culture studies demonstrated that *Spirulina* extracts with 0.1% and 0.05% concentration showed a significant effect on L929 fibroblast cell line proliferation. As fibroblast cells are mesenchymal cells enabling tissue maintenance and support by secreting extracellular matrix, they are responsible for inflammation and scar formation during wound healing. Therefore, wound healing activity on L929 and HS2 cell lines was determined by *in vitro* scratch assay. Results showed that cell proliferation and migration were increased by incorporation of crude extracts. Skin cream incorporated with 1.125% *S. platensis* extract exhibited the highest proliferative effect on skin cells and supported by immunohistochemical assay results. Based on these findings, *Spirulina* incorporation into skin cream or biomaterials will be a promising additive for further use in biomedical applications, particularly as wound dressings.
